# Mechanisms of genotoxicity and proteotoxicity induced by the metalloids arsenic and antimony

**DOI:** 10.1007/s00018-023-04992-5

**Published:** 2023-10-30

**Authors:** Robert Wysocki, Joana I. Rodrigues, Ireneusz Litwin, Markus J. Tamás

**Affiliations:** 1 Department of Genetics and Cell Physiology, Faculty of Biological Sciences, University of Wroclaw, 50-328 Wroclaw, Poland; 2 Department of Chemistry and Molecular Biology, University of Gothenburg, Box 462, 405 30 Göteborg, Sweden; 3 Academic Excellence Hub - Research Centre for DNA Repair and Replication, Faculty of Biological Sciences, University of Wroclaw, 50-328 Wroclaw, Poland

**Keywords:** Arsenic toxicity, Antimony toxicity, Genotoxicity, Proteotoxicity, Proteostasis, Protein folding, Protein aggregation, Metalloid

## Abstract

Arsenic and antimony are metalloids with profound effects on biological systems and human health. Both elements are toxic to cells and organisms, and exposure is associated with several pathological conditions including cancer and neurodegenerative disorders. At the same time, arsenic- and antimony-containing compounds are used in the treatment of multiple diseases. Although these metalloids can both cause and cure disease, their modes of molecular action are incompletely understood. The past decades have seen major advances in our understanding of arsenic and antimony toxicity, emphasizing genotoxicity and proteotoxicity as key contributors to pathogenesis. In this review, we highlight mechanisms by which arsenic and antimony cause toxicity, focusing on their genotoxic and proteotoxic effects. The mechanisms used by cells to maintain proteostasis during metalloid exposure are also described. Furthermore, we address how metalloid-induced proteotoxicity may promote neurodegenerative disease and how genotoxicity and proteotoxicity may be interrelated and together contribute to proteinopathies. A deeper understanding of cellular toxicity and response mechanisms and their links to pathogenesis may promote the development of strategies for both disease prevention and treatment.

## Introduction: chemistry and biology of arsenic and antimony

Arsenic and antimony are classified as metalloids, that is, chemical elements that possess physical and chemical properties that are intermediate between metals and nonmetals. These metalloids are present in trace amounts in the Earth’s crust (mean concentrations: 1–2 ppm for arsenic (13–26 µM) and 0.2 ppm for antimony (1.6 µM)) but can reach high local concentrations due to geological sources or anthropogenic activities [1, 2]. For example, arsenic concentrations in the groundwater from geological sources reaching up to or above 5000 ppb (67 µM) have been measured at sites in Bangladesh, China, Thailand, Argentina, and Australia [3–5], greatly exceeding the levels in drinking water (10 ppb; 0.13 µM) recommended by the World Health Organization (WHO). Similarly, high antimony concentrations have been measured in water and sediments close to mining and smelting areas in China (up to 29,000 ppb; 240 µM), raising concerns that antimony concentrations in the groundwater in these areas may exceed the recommended level in drinking water recommended by WHO (10 ppb; 0.08 µM) [2, 6, 7]

The presence of arsenic in soils and water is a major human health threat, and is estimated to affect up to 200 million people worldwide [8]. Arsenic can accumulate and damage every organ in the human body with the highest concentrations present in the liver and kidney [9]. Chronic arsenic exposure is associated with several pathological conditions including skin disorders, cardiovascular disease, diabetes, cancers, as well as neurological and neurodegenerative disorders [3, 10–13]. Antimony has also been proposed to be a global health threat [7]. Chronic antimony exposure may affect the skin, the respiratory, cardiovascular and gastrointestinal systems, and possibly cause cancer [14, 15]. The highest concentration of antimony in humans is found in liver and kidney, and certain inhaled antimony compounds are retained in lung for long periods [7, 16].

Arsenic concentrations reported in epidemiological studies to cause adverse health effects usually range from 100 to 1000 ppb (1.3–13 µM) and some studies suggest that it may already affect human health below 10 ppb (0.13 µM) [9, 17]. The majority of the cellular studies cited in this review exposed various human cell lines to concentrations ranging from 10 to 100 µM (750–7500 ppb), corresponding to exposure to high environmental arsenic concentrations. Several cellular studies have also used lower arsenic concentrations (from 30 nM up to 10 µM; 5–750 ppb) that correspond to low to moderate environmental arsenic. As a comparison, the median arsenic concentration in healthy humans is in the range of 30–40 ppb (0.4–0.5 µM) in kidney and liver, 50–90 ppb (0.67–1.2 µM) in lung and skin, 10–30 ppb (0.13–0.4 µM) in brain, and 300–900 ppb (4–12 µM) in hair and nail [9]. Tissues taken from arsenic-intoxicated humans show much higher arsenic concentrations: 147,000 ppb (1.9 mM) in liver, 27,000 ppb (360 µM) in kidney, and 11,000 ppb (146 µM) in lung and brain [9]. Mean arsenic concentrations in the plasma and placenta of chronically exposed human populations are about 10 and 100 nM, respectively, while urinary concentrations of exposed individuals can reach up to 5650 ppb (75 µM) [18–21]. Finally, arsenic concentrations in the plasma of individuals treated with arsenic trioxide (As_2_O_3_) peaks at 5–7 µM (400–600 ppb) [22]. Thus, most toxicity mechanisms, both at epidemiological and cellular level, have been studied at moderate to high arsenic concentrations (µM range) while less is known about toxicity targets and mechanisms at low (nM) concentrations.

Epidemiological studies on the health effects of antimony in environmentally relevant concentrations are scarce and mainly concern occupational exposure in industrial settings and during treatment with antimony-containing drugs. The antimony concentration in the lungs of smelter workers exposed to antimony is about 2.6 μM, while the concentration of unexposed individuals is 0.2 μM [16]. The concentration of the pentavalent antimonial drug Glucantime® used to treat leishmaniasis is usually 20 mg/kg body weight per day, and plasma concentrations of antimony peak at 1 mM after injection of the drug [23]. The studies cited in this review usually exposed human cell lines to antimony concentrations ranging from 50 to 800 µM (750–7500 ppb), which corresponds to high exposure levels. Only few studies have explored toxicity mechanisms at low antimony concentrations.

Despite their toxicities, arsenic- and antimony-based drugs have been used for centuries in the treatment of multiple diseases, and current use includes the treatment of acute promyelocytic leukemia (APL) and of diseases caused by certain protozoan parasites; however, the molecular mechanisms of action of arsenic- and antimony-based drugs are in many cases still unclear [10, 24–26]. In spite of its in vitro anticancer activity, arsenic has not been shown to be effective in treating cancer types other than APL in clinical trials [27]. However, its efficacy in anticancer therapy may potentially be increased when combined with other anticancer drugs [28]. Several antimony-based compounds also show marked in vitro antiproliferative activity against a variety of human cancer cells [29, 30]. While antimony-based compounds have not yet been evaluated as anticancer drugs in clinical trials, their lower toxicity compared to arsenic-based compounds illustrates their future potential.

The molecular mechanisms of arsenic and antimony toxicity are only partially understood. In general, toxicity of a metal is governed by its physicochemical properties and ligand preferences. Arsenic and antimony are ‘hard’ transition metals that preferentially interact with oxygen in their higher oxidation states and sulfur in their lower oxidation states. The most common oxidation states are pentavalent and trivalent arsenic and antimony, with the trivalent states being more toxic than the pentavalent states [3, 7, 9, 16]. Typically, metals cause toxicity in vivo by interfering with protein and membrane functions, with nutrient uptake and redox reactions, and by causing DNA damage [31, 32]. At the cellular level, arsenic toxicity has been attributed to oxidative stress, changes to the epigenome, genotoxicity, and to altered protein function and activity. At the molecular level, pentavalent arsenate [As(V)], being chemically similar to phosphate, can cause toxicity by competing with phosphate in various biochemical reactions and by impairing energy generation via disruption of adenosine triphosphate (ATP) production. The toxicity of trivalent arsenite [As(III)] is mainly attributed to its high affinity for sulfhydryl groups, and binding of As(III) to reduced cysteine residues in proteins may disturb protein conformation, function, and interactions [32–36]. The ability of As(III) to bind to proteins is not only central for its toxic mode of action but can also contribute to its therapeutic effects. For example, binding to cysteine residues in the oncoprotein PML-RARα underlies the anticancer activity of arsenic trioxide in patients with APL [37, 38]. Similarly, As(III) binding to specific kinases and transcriptional regulators is associated with enhanced arsenic resistance in yeast and bacteria [39–41].

Compared to arsenic, much less is known about the mechanisms of antimony toxicity. It is generally assumed that arsenic and antimony are structurally related, and that they therefore act in similar ways. Like As(III), antimonite [Sb(III)] has been proposed to affect the cellular redox balance, to disrupt enzyme function by binding to sulfhydryl groups in proteins, and to cause DNA damage [15, 42–44]. Additionally, Sb(III) binding to proteins is not only associated with toxicity but also with antimony resistance in yeast and bacteria [39, 45, 46].

Metalloid toxicity is also dependent on metabolic transformations. Arsenic and antimony can undergo biomethylation in many microorganisms and mammalian species, including humans. However, the extent of biomethylation varies between species and may also give rise to species-specific compounds [44, 47, 48]. In humans, arsenic methylation primarily occurs in the liver and involves the arsenic (+ 3 oxidation state) methyltransferase enzyme As3mt [48]. Whether antimony is methylated in humans is unclear [44]. Nevertheless, antimony can be methylated in vitro by the human As3mt orthologue ArsM, isolated from the red alga *Cyanidioschyzon merolaeis* [49], raising the possibility that methylation might occur also in humans. Methylation changes the properties and behavior of arsenic and antimony and affects their modes of action and toxicities with methylated forms considered to be more toxic than the inorganic forms [48]. One way methylation affects metalloids is by modulating their binding to proteins: inorganic As(III) can bind up to three cysteine residues, monomethylarsonous acid [MMAs(III)] can bind two cysteine residues and dimethylarsonous acid [DMAs(III)] can bind only one cysteine residue [33]. Studies in budding yeast (*Saccharomyces cerevisiae*), that possesses a methyltransferase enzyme involved in As(III) methylation [50], indicated that As(III) methylation also serves a signaling function in an adaptative response to arsenic stress [50, 51]. Thus, depending on the form of the metalloid and on the specific target, arsenic and antimony binding to proteins may cause toxicity or resistance.

Studies in the past decades have greatly expanded our mechanistic understanding of arsenic and antimony toxicity and emphasized genotoxicity and proteotoxicity as key contributors to pathogenesis. Our understanding of how cells resist and adapt to metalloid toxicity has also increased substantially in recent years. In this review, we highlight mechanisms by which arsenic and antimony cause genotoxicity and proteotoxicity as well as mechanisms used by cells to maintain proteostasis during metalloid exposure. We also address how metalloid-induced proteotoxicity may promote neurodegenerative disease and how genotoxicity and proteotoxicity may be interrelated and contribute to proteinopathies in a concerted way.

## How arsenic and antimony cause genotoxicity

Over the past decades, a large number of epidemiological studies documented a strong association between environmental, occupational, and medical exposure to arsenic and cancers of the skin, lung, and urinary bladder [52, 53]. Arsenic exposure has also been linked to the development of kidney, liver, and prostate cancer [53–56]. Consequently, arsenic, and its inorganic compounds, is classified as a group 1 human carcinogen [53]. Paradoxically, arsenic exhibits a weak mutagenic potential, even at toxic concentrations, and its carcinogenic properties failed to be reproduced in experimental animals for many years, obscuring its mechanisms of carcinogenesis [57, 58]. More recent studies, using transplacental/early-life or whole-life exposure approaches, found that inorganic arsenic has the ability to induce several types of cancer in mice [58–60]. Additionally, arsenic is a well-established co-carcinogen that potentiates the carcinogenic effects of ultraviolet radiation and benzo[a]pyrene present in tobacco smoke, and enhances the mutagenicity and clastogenicity of known genotoxins, such as cisplatin, methyl methanesulfonate, *N*-methyl-*N*-nitrosourea, and phleomycin [17, 61, 62]. Several, mutually non-exclusive, mechanisms of arsenic-elicited carcinogenesis have been proposed, reflecting arsenic’s multifaceted effects at the cellular level: these include (a) induction of oxidative stress resulting in increased oxidative DNA damage; (b) biotransformation of inorganic arsenic to methylated forms with higher genotoxicity; (c) inhibition of DNA repair mechanisms via transcriptional repression of genes encoding DNA repair enzymes and by modulating their enzymatic activity; (d) non-genotoxic epigenetic dysregulation (DNA methylation, post-translational histone modifications, miRNA) and aberrant activation of signal transduction pathways, which lead to expression changes of tumor suppressor genes and proto-oncogenes at the level of transcription initiation and alternative splicing, ultimately resulting in stimulation of cell proliferation and inhibition of apoptosis [17, 52, 63, 64]. Moreover, studies in budding yeast indicated that arsenic can generate DNA damage independently from oxidative stress, suggesting that additional modes of arsenic genotoxicity exist [62].

The genotoxic and carcinogenic potential of antimony is much less studied. The few available epidemiological studies of occupational and environmental exposure to antimony found either no association with cancer incidence or an increased risk of lung cancer [44, 65]. However, a rodent inhalation study revealed that Sb(III), in form of antimony trioxide, has the potential to induce lung and adrenal gland tumors as well as lymphomas [66]. Consequently, Sb(III) is currently categorized as probably carcinogenic to humans (group 2A) [67]. Like for arsenic, the carcinogenic properties of Sb(III) may result from induction of oxidative stress, inhibition of DNA repair mechanisms and changes in gene expression, leading to increased oxidative DNA damage and stimulation of cell proliferation [44]. Additionally, studies in *S. cerevisiae* revealed that Sb(III) also disrupts telomere maintenance and causes topoisomerase I (Top1)-dependent replication-associated DNA damage [68].

Up to now, the molecular mechanisms of metalloid-induced DNA damage and carcinogenesis remain elusive and still under debate. This section will highlight how As(III) and Sb(III) cause genotoxicity by partly distinct mechanisms.

### Arsenic and antimony-induced genotoxic effects in mammalian cells

**Fig. 1:**
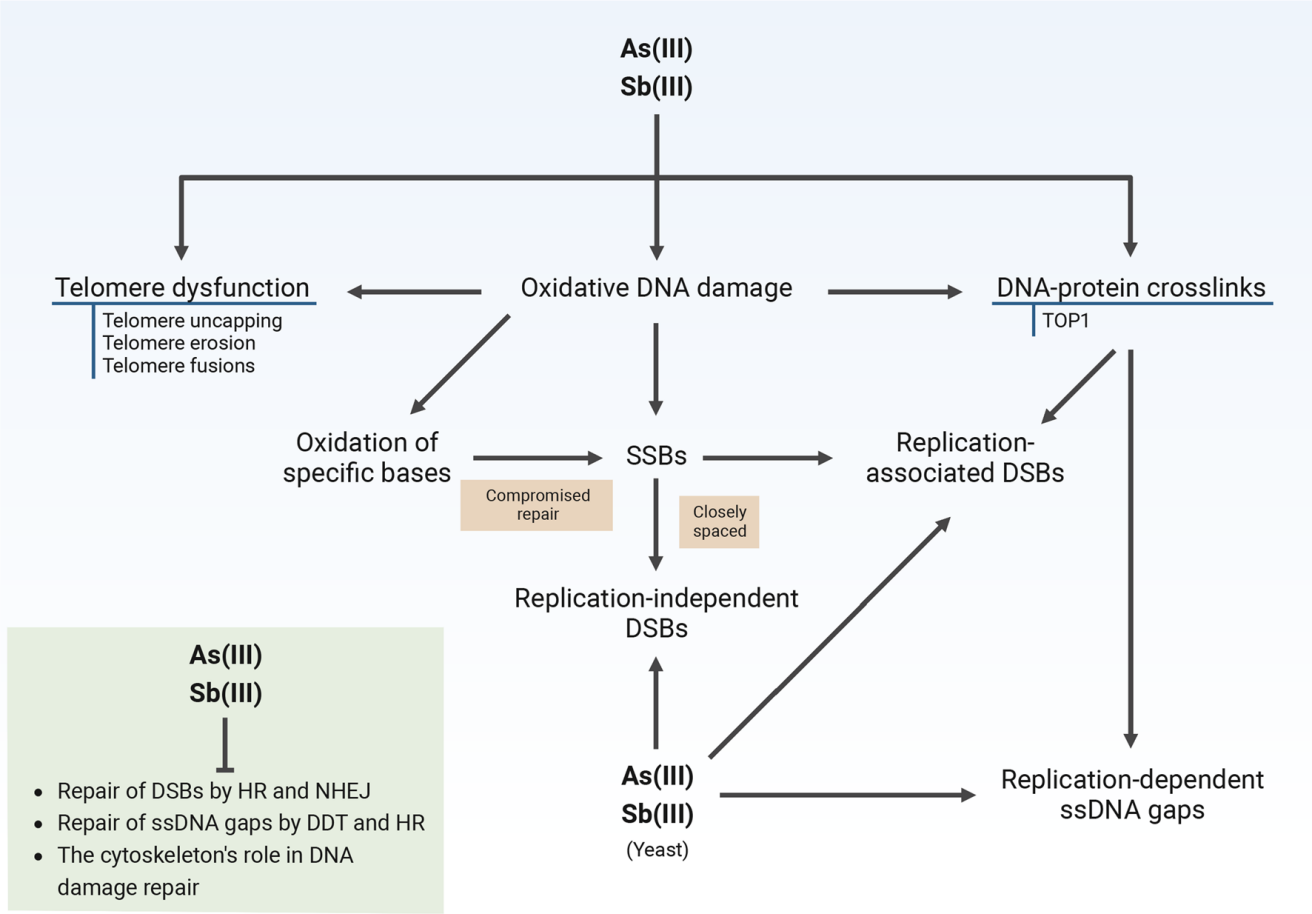
How arsenic and antimony cause genotoxicity. Both in yeast and mammalian cells, As(III) and Sb(III) induce oxidative stress to varying degrees. This results in elevated levels of oxidative DNA damage, including oxidized bases and single-strand breaks (SSBs), which can also arise indirectly from incomplete repair of oxidized bases by base excision repair. SSBs can be converted to double strand breaks (DSBs) during replication or when SSBs are closely spaced. In addition, As(III) and Sb(III) increase the formation of protein–DNA adducts such as topoisomerase 1 (TOP1) DNA–protein crosslinks, either in a DNA oxidative damage-dependent manner or by interfering with TOP1 enzymatic activity. The presence of DNA–protein crosslinks leads to generation of replication-associated DSBs and single-stranded DNA (ssDNA) gaps. In budding yeast, oxidative stress and replication-independent DNA damage were also observed after metalloid treatment. In addition, As(III) and Sb(III) not only induce DSBs and ssDNA gaps, but also inhibit repair of these lesions by interfering with DNA damage repair pathways such as homologous recombination (HR), non-homologous end joining (NHEJ) and DNA damage tolerance (DDT). As(III) and Sb(III)-mediated disruption of the actin and microtubule cytoskeleton may also interfere with various aspects of DNA damage repair and cause chromosome aberrations. Finally, both metalloids perturb homeostasis of telomeres by oxidation of guanine-rich telomeric repeats, possibly by directly binding to telomeric DNA or by interfering with the function of telomere-associated proteins. This leads to telomere uncapping resulting in telomere erosion and fusion of chromosome ends. The figure was created with BioRender.com

As(III) is inactive or extremely weak in its ability to induce gene mutations [63]. Moreover, there is no solid evidence for the reactivity of As(III) with DNA. However, it is well known that in cultured human cells, long-term exposure to As(III) at moderate to high concentrations (≥ 5 μM for 16–24 h) causes oxidative modifications of DNA and oxidative damage-related lesions, such as 8-hydroxyl-2′-deoxyguanine (8-OHdG), DNA breaks and DNA–protein crosslinks, as well as sister chromatid exchanges and chromosomal aberrations, including micronuclei and aneuploidy [52, 63]. Accordingly, several studies showed that As(III) increases production of reactive oxygen species (ROS) and reactive nitrogen species (RNS), whereas As(III)-induced oxidative DNA damage can be attenuated by the addition of antioxidants [69, 70]. Elevated levels of urinary 8-OHdG in individuals chronically exposed to high As(III) concentrations further supports the notion that high doses of As(III) cause DNA damage by increasing oxidative stress [71] (Fig. 1). Interestingly, induction of ROS production and increased oxidative DNA damage was also observed in the breast cancer cell line MCF-7 exposed to non-cytotoxic concentrations of As(III) (2 μM for 4 h) [72]. However, identical conditions did not increase ROS production in mouse embryonic fibroblasts (3T3) and three human cell lines (HeLa, HEK 293, HEMn-LP), providing evidence against the oxidative stress theory of arsenic carcinogenesis during chronic exposure to low levels [73].

Oxidation of bases in DNA and single-strand breaks (SSBs) are formed directly from hydroxyl radical attack. Additionally, SSBs can be generated during the repair of oxidized bases by base excision repair (BER). Furthermore, replication of DNA containing unrepaired SSBs results in the formation of double-strand breaks (DSBs) leading to chromosomal aberrations and genomic instability. Indeed, As(III) at concentrations of 5–50 μM induced replication-dependent DSBs in cultured AA8 Chinese hamster ovary cells, which activated the DNA damage response (DDR) followed by homologous recombination (HR) repair [74]. Moreover, in HR-deficient cells, DSBs were detectable already at 1 μM As(III) [74]. Additionally, closely spaced SSBs can also generate DSBs in the absence of replication, but this requires very high levels of oxidative damage and/or region-specific accumulation of such lesions. How As(III) generates oxidative stress-dependent DNA damage is not well understood. Metalloids are not redox-active elements, like iron (Fe) or copper (Cu), so it seems that they must induce ROS production indirectly. Early studies showed that ROS generated in As(III)-treated cells are mainly produced by membrane-bound NADPH oxidase (NOX), due to expression upregulation of NOX complex components [75, 76]. Interestingly, depletion of NOX components in leukemic cells led to nearly complete inhibition of ROS production by As(III) [76]. Other studies showed that As(III) at the concentrations of 15–10 µM also targets mitochondria, leading to the release of the superoxide anion and its subsequent reaction with nitric oxide to produce the less reactive and more stable peroxynitrite, which has the capacity to act throughout the cell [77, 78]. 2 μM As(III) was also able to increase the levels of nitric oxide by upregulating the expression of nitric oxide synthase (NOS) in human keratinocytes [79]. Other types of ROS generated by As(III) via NOX or mitochondria, such as H_2_O_2_, are too reactive and unstable to penetrate the nucleus and induce oxidative DNA damage [80]. Methylated forms of arsenic are a potential source of ROS that can be generated within the nucleus. It has been demonstrated that the reaction between molecular oxygen and DMAs(III) produces dimethylarsenic peroxyl radical and superoxide anion radical [81]. Importantly, methylation of As(III) and subsequent generation of dimethylarsenic peroxyl radical likely contributes to induction of DNA breaks in lung cells of rodents and human alveolar epithelial type II (L-132) cells [82]. Finally, in human CD4 T lymphocytes, As(III) at the cytotoxic concentration of 5 μM has recently been shown to induce the accumulation of topoisomerase 1-DNA covalent cleavage complexes (TOP1cc) [83], which, in turn, can stall replication fork progression leading to generation of DSBs. Importantly, TOP1cc can be trapped on DNA due to inhibition of TOP1 activity or by various DNA lesions, including oxidative DNA damage [84].

Another source of DNA damage, such as chromosomal aberrations and aneuploidy in cells exposed to As(III), may be dysfunctional telomeres (Fig. 1). Epidemiological studies reported both lengthening and shortening of telomeres in individuals chronically exposed to As(III) [85]. A possible mechanism for As(III)-induced telomere lengthening involves the upregulation of the telomerase catalytic subunit (hTERT) and telomere binding proteins such as TRF1 and TRF2, subunits of the telomere sheltering complex [85]. Thus, As(III) may partially contribute to carcinogenesis by promoting telomere elongation, which is crucial for the maintenance and viability of human cancer cells. Paradoxically, As(III)-induced downregulation of both hTERT and TRF2 was also observed and associated with telomere shortening. Since telomere sequences are rich in guanine, which is particularly sensitive to oxidative damage, it has been proposed that As(III) may cause telomere attrition through oxidative stress [85]. Importantly, 4 μM As(III) induced increased ROS levels in glioblastoma cells (U87 line) followed by translocation of telomerase from the nucleus to the cytoplasm, telomere shortening, and accumulation of γH2AX (histone H2AX variant phosphorylated on Ser139, which serves as a sensitive marker of DNA damage and repair) that co-localized with TRF1, strongly suggesting that the telomeres are sites of As(III)-generated DNA damage in an oxidative stress-dependent manner [86]. Activation of DDR at telomeres and erosion of telomeres accompanied by downregulation of TRF2 expression were also observed in human CD4 T lymphocytes exposed to 5 μM As(III) [83]. A study in CD1 mouse embryos revealed that 30 μM As(III) induced telomere erosion and end-to-end chromosome fusions, which was prevented by co-administration of an antioxidant [87]. Interestingly, telomerase-deficient mouse embryos with shortened telomeres exhibited decreased resistance to As(III) [87]. Moreover, prostate cancer cells (PC-3 line) characterized by very short telomeres were about 10- to 100-fold more sensitive to As(III) compared to human cell lines with longer telomeres [88]. Another study also reported an association between longer telomere lengths and increased resistance to As(III) in human cells in vitro [89]. In PC-3 prostate cancer cells, cytotoxic concentrations of As(III) (0.23–0.45 μM) triggered telomere dysfunction, manifested by telomere-associated accumulation of γH2AX and telomere degradation, possibly by directly binding to telomeric TTAGGG repeats, resulting in displacement of hTERT from telomeres [88]. Importantly, telomeric DNA damage was observed in the absence of oxidative DNA damage in PC-3 cells, suggesting that oxidative stress-independent telomere dysfunction may be a key source of As(III)-induced genotoxicity in cells with short telomere length [88].

Unlike for As(III), there are limited data on the genotoxic potential of Sb(III). Sb(III) did not induce mutations either in bacterial assays or in cultured mammalian cells [44], although one early report showed a positive response to SbCl_3_ in the *Bacillus subtilis* Rec-assay [90]. Many studies in mammalian cell cultures or rodents have shown no clastogenic effects of Sb(III) [44, 68]. Moreover, 50 μM SbCl_3_ did not induce any detectable DSBs in HeLa S3 cells after 8 h treatment [91]. On the other hand, exposure of HepG2 and LS-174 T human cancer cell lines to high concentrations of SbCl_3_ (100–500 μM) resulted in the induction of the DNA damage marker γH2AX [92]. Some historical studies reported clastogenic activity of Sb(III), such as induction of micronuclei, sister chromatid exchange and chromosomal aberrations [44, 68]; however, these studies have recently been inspected according to the applicable standards and have turned out to be mostly inconclusive and of poor quality [44]. A more recent analysis of the genotoxic properties of antimony compounds by the ToxTracker assay revealed that low concentrations of Sb(III) (0.2–0.5 μg/mL or 0.3–0.8 μM) have the potential to induce oxidative stress-derived DNA damage but has no ability to directly damage DNA or interfere with replication [93]. 1 μg/mL (8 µM) of Sb(III) also activated the unfolded protein response suggesting induction of protein stress [93]. Very high concentrations of Sb(III) (105 μg/mL; 860 μM) strongly disrupted redox homeostasis in human THP-1 macrophages, leading to a 50% decrease in intracellular free glutathione (GSH) levels, due to the formation of Sb(GS)_3_ conjugates as a mean of Sb(III) detoxification, and due to Sb(III)-mediated inhibition of glutathione reductase catalyzing the regeneration of GSH from oxidized GSSG [94]. SbCl_3_ at the concentration of 60 μM was demonstrated to cause death of rat hepatocytes by generating ROS [95]. Furthermore, in zebrafish liver, high concentrations of Sb(III) (16.58 and 33.16 mg/L) induced oxidative stress resulting in the accumulation of oxidative DNA damage but not DNA–protein cross-links [96]. In summary, Sb(III) appears to exhibit an indirect genotoxic mode of action involving increased oxidative stress at relatively high concentrations, but further research is needed to firmly establish Sb(III) as a genotoxin for animals, including humans.

### Studies of arsenic and antimony genotoxicity in budding yeast

*S. cerevisiae* is an excellent model organism to study various aspects of DNA damage response, DNA repair pathways, and mechanisms of DNA damage induction by chemical agents [97–99]. Studies using either wild-type yeast or mutants devoid of As(III)/Sb(III) detoxification transporters Acr3 and Ycf1 revealed that both As(III) and Sb(III) exhibit genotoxic properties at subcytotoxic concentrations [62, 68]. 1 h exposure to As(III) triggered phosphorylation of histone H2A on Ser129 (an equivalent of human γH2AX, which is a sensitive DNA damage marker also in yeast) [97–99], starting from 250 μM in wild type and 50 μM in the double mutant *acr3*Δ *ycf1*Δ [62, 68]. For Sb(III), high levels of H2A phosphorylation were observed after 1–2 h treatment with 200–500 μM in *ycf1*Δ cells [62, 68]. In contrast to mammalian cells, subcytotoxic or cytotoxic concentrations of both As(III) (0.5–1 mM) and Sb(III) (0.2–5 mM) induced low levels of ROS and only a small increase in oxidative DNA damage [62, 68]. Moreover, a BER-defective mutant showed a very mild sensitivity to either metalloid, strongly suggesting that As(III) and Sb(III) have weak potential to generate oxidative stress-dependent DNA damage in budding yeast. Also, cells defective in nucleotide excision repair (NER) showed wild type resistance to both metalloids, which indicates that As(III) and Sb(III) do not generate bulky DNA adducts in yeast. In contrast, HR- and DNA damage tolerance (DDT)-defective yeast mutants devoid of DSB and replication-associated damage repair, respectively, were substantially As(III) and Sb(III) sensitive. Consequently, As(III) and Sb(III)-treated S phase cells showed increased accumulation of SSBs and ssDNA gaps, as well as Rad52 DNA repair nuclear foci, which indicate ongoing DNA repair by HR [62, 68] (Fig. 1). For Sb(III), the levels of SSBs, ssDNA regions and Rad52 foci were partially reduced in cells lacking Top1, but not by the addition of a ROS scavenger [68]. This indicates that a subset of Sb(III)-induced replication lesions may be generated due to the formation of Top1-DNA adducts, but independently from oxidative stress (Fig. 1). Interestingly, induction of Top1-induced DNA fragmentation by As_2_O_3_ (1–2 μM for 72 h) has also been reported in human leukemia NB4 cells, but in an oxidative stress-dependent manner and accompanied by apoptosis activation [100].

Sensing of DSBs or ssDNA gaps by the upstream kinases of the DDR, Mec1 and Tel1, results in phosphorylation of histone H2A and hyperphosphorylation of the effector kinase Rad53, which serve as sensitive markers for these types of DNA damage [97]. Importantly, both As(III) and Sb(III) induce high levels of H2A and Rad53 phosphorylation, not only in S phase but also in G_2_/M phase, suggesting DSB induction in the absence of replication and elevated oxidative stress (Fig. 1). In G_1_ phase-synchronized cells devoid of the Ku complex, that binds to and protects DSBs from resection and thus prevents activation of DDR in G_1_, H2A and Rad53 phosphorylation was also observed after As(III) and Sb(III) treatment. However, the presence of DSBs was detectable only at a very high concentration of As(III) (25 mM) [62, 68]. Thus, it remains to be established what types of DNA lesions are mainly responsible for As(III)-induced activation of DDR in yeast at subcytotoxic concentrations. Moreover, no presence of DSBs was detected upon Sb(III) treatment of yeast cells [68]. Instead, Sb(III) negatively affects telomere stability, since mutants devoid of proteins involved in telomere maintenance, such as Cdc13, Tel1, and Yku70, were found to be highly sensitive to Sb(III) [68]. Importantly, Sb(III) sensitivity of the *cdc13-1* mutant was strongly supressed by deletion of the *PIF1* or *EXO1* genes encoding nucleases contributing to resection of uncapped telomeres. The *cdc13-1* mutant also showed high sensitivity to As(III), which suggests that both metalloids are able to disrupt homeostasis of telomeres leading to DDR activation [68] (Fig. 1). The fact that metalloid-induced DNA damage in yeast cells is largely independent of oxidative stress and also observed in the absence of replication, suggests that both As(III) and Sb(III) might also exhibit in situ DNA damage activity. We hypothesize that both metalloids might generate low levels of sequence- or chromosome site-specific DSBs, which are not detectable by standard techniques.

However, the question remains whether the mechanisms of As(III) and Sb(III) genotoxicity observed in budding yeast may be similar in human cells. Compared to mammals, yeast cells are more resistant to both metalloids, in part due to the presence of efficient detoxification pathways mediated by the plasma membrane As(III)/Sb(III) efflux transporter Acr3 and the ATP-binding cassette (ABC) transporter Ycf1, which sequesters As(III)/Sb(III)-glutathione conjugates into the vacuole [101]. Indeed, the use of yeast mutants lacking these transporters allowed to decrease concentrations of As(III) and Sb(III) exerting genotoxic effects by five- and tenfold, respectively [62, 68]. Members of the Acr3 family are absent in animals; however, mammalian cells express a wide plethora of plasma membrane-localized ABC transporters, which pump free or glutathione-conjugated arsenic species, including inorganic As(III), MMAs(III) and dimethylarsinic acid [DMAs(V)] out of the cells, enabling arsenic clearance from the whole body [101]. Upregulation of these transporters is the key mechanism of acquired high-level As(III) resistance observed in cancer cells [101]. Both yeast and mammals can also limit intracellular accumulation of As(III) and Sb(III) by downregulating expression or activity of aquaglyceroporins and sugar transporters, which serve as entry pathways for As(III) and Sb(III) in all kingdoms of life [101]. On the other hand, these channels and transporters were also shown to extrude methylated forms of arsenic in mammalian cells. Interestingly, it has been recently demonstrated that budding yeast also produce MMAs(III) and DMAs(III), which contribute to the toxic effects of inorganic As(III) exposure [50, 51]. Whether the production of methylated As(III) species increases DNA damage in yeast cells remains to be investigated. Higher resistance of yeast cells to As(III) and Sb(III) compared to human cells may also be explained by the fact that budding yeast lacks certain DNA repair proteins and cycle regulators, such as the poly(ADP-ribose) polymerase-1 (PARP-1) protein, BRCA1 and p53, which are known to be targeted by these metalloids (see below). On the other hand, Chinese hamster ovary (CHO-K1) cells, often used in metalloid genotoxicity studies, are about 10 times more resistant to As(III) than human fibroblasts [102]. Even human cell lines can vary greatly in susceptibility to As(III) [88]. Differences in arsenic resistance between animal species may be due to the length of telomeres [87], antioxidant capacity [102] and dissimilar rates and patterns of arsenic methylation [103, 104]. Nevertheless, it should be emphasized that DNA damage caused by As(III) and Sb(III) in all model organisms and cell lines is observed mainly at species-appropriate subcytotoxic and cytotoxic concentrations and it remains to be determined whether or how the genotoxic potential of As(III) and Sb(III) contributes to the development of cancer in humans during chronic exposure to low levels of metalloids.

### Inhibition of DNA repair by arsenic and antimony

It is well documented that low concentrations of As(III) potentiates DNA damage induced by several physical and chemical agents in mammalian cells via indirect or direct inhibition of DNA repair pathways, and thus may contribute to genomic instability and, ultimately, cancer (Fig. 1). A number of studies reported that As(III) treatment results in decreased expression of several DNA repair genes involved in BER and NER, as observed in mammalian cell lines and in cells isolated from individuals chronically exposed to As(III) [17, 105]. As(III)-induced downregulation of gene expression likely involves changes in DNA methylation and histone modification patterns. For example, 1 μM As(III) reduces expression of the DSB repair *BRCA1* gene by inducing hypermethylation of its promoter in MCF7 breast cancer cells [106], while dose-dependent decrease in expression of several BER genes (*MPG*, *PARP1*, *XRCC1*) in human keratinocytes (HaCaT cells) treated with subcytotoxic and cytotoxic concentrations of As(III) (2.5–10 μM) was associated with the H3K9me2 heterochromatin mark enrichment in their promoter regions [107]. Decreased expression of some mismatch repair (MMR) genes, such as *MLH1* and *MSH2*, due to promoter DNA hypermethylation was observed in HaCaT cells treated with 0.1–0.5 μM As(III) [108] and in peripheral blood mononuclear cells isolated from humans chronically exposed to As(III) in drinking water [109].

As(III) can also directly impact the activity of DNA repair proteins. As(III) has been shown to bind to zinc finger (ZnF) motifs, which are common in DNA-binding proteins, including transcription factors and DNA repair proteins, as well as in so-called Really Interesting New Gene (RING) domain proteins such as E3 ubiquitin ligases [110]. Mass spectrometry analysis revealed that As(III) selectively displaces Zn(II) from C3H1 and C4 ZnF motifs, but not from the more common C2H2 motif, and that As(III) is coordinated by three cysteine residues [111]. In contrast, MMAs(III) binds to two cysteines and can thus interact with all types of ZnF motifs [112]. Arsenic-bound ZnF motif-containing peptides display an altered conformation, suggesting that As(III) binding may affect the function of ZnF proteins [113]. Supporting this view, in vivo assays indicated that very low concentrations (0.01–2 μM) of As(III) and MMAs(III) inhibit the activity of PARP-1, which contains two C3H1-type ZnFs [111, 114–116]. Interestingly, it has been suggested that As(III) binding to the ZnFs of PARP-1 per se does not exert an inhibitory effect: instead, the As(III)-bound cysteines in PARP-1 are highly susceptible to oxidation by ROS, which ultimately leads to its inactivation [117, 118]. In addition, As(III)-induced ROS or RNS may also be responsible for oxidation/*S*-nitrosylation of PARP-1 resulting in Zn(II) loss and inhibition of its activity [119, 120]. PARP-1 acts as a sensor of SSBs and DSBs, which stimulate PARP-1-mediated synthesis of poly(ADP-ribose) (PAR) and the attachment of PAR chains onto itself and other proteins involved in the cellular response to DNA damage and DNA metabolism. The presence of PAR chains at the site of DNA damage facilitates repair processes by chromatin relaxation and by recruiting proteins involved in SSB repair, removal of bulky adducts by NER, removal of abortive TOP1cc, and DSB repair by HR and non-homologous end-joining (NHEJ) [121]. Inhibition of PARP-1 activity by 2 μM As(III) strongly perturbs the repair of H_2_O_2_- and ultraviolet radiation-induced DNA damage in human keratinocytes (HaCaT cells) [115, 116, 122]. Another function of PARP-1 is to downregulate repair of DSBs by HR and therefore, PARP-1 inhibition increases the level of sister chromatid exchanges [121], which is a hallmark of genomic instability frequently observed in human cells upon As(III) exposure [123]. Importantly, mouse embryonic fibroblasts lacking PARP-1 exposed to 11.5 μM As(III) exhibited elevated levels of DNA damage, micronuclei induction, telomere attrition and increased cell death [124]. This suggests that, in addition to inhibiting DNA repair, As(III) may itself induce DNA lesions that are repaired by PARP-1-dependent pathways.

The xeroderma pigmentosum complementation group A (XPA) protein, which contains the C4-type ZnF, is another As(III)-targeted DNA repair protein [111, 122]. XPA plays a central role in NER by coordinating the assembly of core NER factors at the site of DNA damage [125]. Initially, it was suggested that As(III) does not interfere with the binding of XPA to UV-irradiated oligonucleotides [126]. Another group reported that As(III) disrupts the binding of XPA to DNA oligomers crosslinked with mitomycin C only at very high concentrations (0.5–2 mM) [127]. Moreover, MMAs(III) was found to exhibit much higher affinity for the XPA ZnF compared to As(III) [128]. However, more recent reports demonstrated that As(III) at the concentrations of 0.5–2 μM was able to decrease XPA binding to chromatin in human keratinocytes (HEKn cells) [122] and XPA and PARP-1 displayed similar affinities for As(III) [129].

Finally, As(III) can bind to the RING finger domain of E3 ubiquitin ligases, such as RNF20–RNF40, FANCL, and RAD18, leading to inhibition of DSB repair, DNA interstrand crosslink repair (ICR), and replication bypass of UV-induced DNA lesions by translesion synthesis (TLS), respectively [130–132]. This suggests that As(III) may interfere with the function of RING-type E3 ubiquitin ligases in the cell, possibly disrupting not only DNA repair pathways but also ubiquitin-dependent protein degradation. As(III) binding to ZnF proteins may also stimulate their degradation, as shown for the histone acetyltransferase TIP60 in human HEK293T cells treated with 2–5 μM As(III) [133] and the oncogenic PML-RARα fusion protein in APL cells at the therapeutic concentration of 1 μM [37]. Notably, TIP60 activity is not only important for gene regulation but also for facilitating DSB repair by HR [134].

Less is known about the effect of Sb(III) on DNA repair. In A549 human lung adenocarcinoma cells, SbCl_3_ at subcytotoxic and cytotoxic concentrations (0.25–0.5 mM) was shown to specifically inhibit the repair of ultraviolet C (UVC) radiation-induced cyclobutane pyrimidine dimers by NER. This was associated with reduced expression of the NER protein XPE and the displacement of Zn(II) from XPA, which resulted in decreased binding of XPA to chromatin [135]. In vitro, Sb(III) binds to a CCCH-type peptide with high affinity, but also to CCHC-type ZnF peptides [136, 137]. Non-cytotoxic concentrations of Sb(III) (1–8 μM) have also been shown to trigger degradation of the RING finger PML-RARα oncoprotein in the APL cell line NB4 [138, 139]. Thus, similar to As(III), Sb(III) may have the potential to inhibit C3H1 and C4-type ZnF proteins involved in DNA repair. In line with this notion, an early report demonstrated that high concentrations of Sb(III) (0.2–0.4 mM) significantly inhibited DSB repair in Chinese hamster ovary cells (CHO-K1) [140].

More recently, it has been shown that non-cytotoxic concentration of Sb(III) (10 μM) also inhibited the repair of radiation-induced DSBs in human HeLa S3 cells in all phases of the cell cycle, suggesting that Sb(III) impairs DSB repair by both NHEJ and HR [91]. Importantly, Sb(III) did not interfere with the initial steps of DDR, but decreased the recruitment of the HR repair proteins BRCA1 and RAD51 to sites of DSBs [91]. BRCA1 is likely a direct target of Sb(III) due to the presence of a RING domain within BRCA1, and since As(III) has been reported to release Zn(II) from BRCA1 [132]. 0.2 mM Sb(III) also inhibited DSB repair in budding yeast by impairing NHEJ-dependent processes such as fusion of unprotected telomeres and re-joining of a linearized plasmid, and by negatively affecting the repair of phleomycin-induced DSBs [68].

### The cytoskeleton as a target for arsenic and antimony cytotoxicity

It is well established that As(III) can cause aneuploidy, which is a driving force in many types of cancer [141, 142]. Early reports suggested that As(III) may induce chromosome missegregation by disrupting the mitotic spindle. 16 h exposure of mouse fibroblasts (Swiss 3T3 line) to high concentrations of As(III) (20 μM) resulted in morphological loss of microtubules [107], while low concentrations of As(III) (0.01–0.1 μM for 24 h) distorted the microtubules of the spindle apparatus and induced aneuploidy in human lymphocyte cultures [143–145]. As(III) also inhibited guanosine triphosphate (GTP)-induced polymerization of tubulin at 1 mM, while MMAs(III) and DMAs(III) caused inhibition at 10 μM, as demonstrated by the acellular tubulin assembly assay [146]. A more recent study reported that 20–40 μM As(III) inhibited the in vitro assembly of microtubules and a significant number of rat lung fibroblasts (RFL6 cell line) exposed to 10 μM As(III) for 24 h exhibited damage to spindle microtubules [147]. In mammalian cells, As(III) has been proposed to interfere with tubulin polymerization and microtubule formation by binding to two vicinal cysteine residues (Cys12 and Cys213) within the β-tubulin monomer, thereby preventing GTP binding, which is indispensable for the formation of tubulin polymers [145]. In contrast, G_2_ phase-enriched human fibroblasts exposed to 5 μM As(III) for 24 h exhibited derangement of the spindle apparatus and chromosome loss, but no inhibition of spindle formation was observed [148]. Similarly, another study found that disruption of mitosis by 5 μM As(III) in three human cell lines was not dependent on direct inhibition of tubulin polymerization but instead involved heat shock-like perturbation of centrosome function, resulting in centrosome fragmentation, and multi-polar spindle and mitotic arrest [149]. In budding yeast, subtoxic concentrations of As(III) [150, 151] and Sb(III) [68] also distort the morphology of the microtubule cytoskeleton, likely by inhibiting the chaperonin TRiC/CCT complex required for tubulin folding but not by inhibiting tubulin polymerization [151]. However, it is important to emphasize that yeast tubulin lacks the Cys12 residue, which was proposed to be targeted by As(III) in human tubulin. Perturbation of the spindle assembly checkpoint (SAC) resulting in metaphase arrest bypass is another possible mechanism of As(III)-induced aneuploidy [141]. For example, arsenic-induced squamous cell carcinomas are characterized by increased expression of microRNA-186, which negatively modulates expression levels of SAC regulators [152]. Moreover, human keratinocytes overexpressing microRNA-186 exhibited increased levels of aneuploidy during chronic exposure to low concentrations of As(III) (100 nM for 8 weeks) [152, 153].

Like tubulin, actin has been identified as an As(III)-binding protein in human cells [145, 154]. As(III)-induced disruption of the actin cytoskeleton was demonstrated at 2.5 μM in mouse fibroblasts (Swiss 3T3 line) [143–145], 5 μM in human acute leukemia cells (HL-60 line) [155] and 10 μM in mouse endothelial cells (SVEC4-10 line) [156]. In budding yeast, both As(III) [150, 151] and Sb(III) [68] at subtoxic concentrations also triggered reorganization of the actin cytoskeleton. Similar to tubulin, it has been suggested that As(III) interferes with actin folding by inhibiting the chaperonin TRiC/CCT complex [151]. Interestingly, other stress conditions, including oxidative stress, cause similar rearrangements of the actin cytoskeleton that may result from the formation of an intramolecular disulfide bond between conserved Cys374 and Cys285 residues or an intermolecular disulfide bond with Cys374 of another actin molecule [157, 158]. Importantly, the yeast *act1-Cys285A,C374A* mutant failed to reorganize the actin cytoskeleton in response to acute oxidative stress and exhibited increased sensitivity to oxidative stress [157]. This suggests that stress-induced rearrangement of the actin cytoskeleton may serve as an adaptive/protective stress response. Indeed, in yeast cells, As(III)-induced depolarization of the actin cytoskeleton was transient, followed by a recovery of the polar actin distribution accompanied by resumption of growth [150, 151]. The phenotype of yeast cells expressing the mutant actin lacking Cys285 and Cys374 in the presence of As(III) and Sb(III) has not been reported.

There is increasing evidence pointing to an important contribution of nuclear actin filaments in DSB repair by modulating chromosome movements and clustering of repair sites [159]. On the other hand, the interphase cytoplasmic microtubule network participates in trafficking of DNA repair factors to the nucleus [160] and facilitates DNA repair by promoting changes in nuclear morphology and chromatin organization as well as mobility of DNA lesions in mammalian cells [161]. Whether As(III) and Sb(III) exert their negative effects on DNA repair by disrupting nuclear actin filaments or interphase cytoplasmic microtubules, remains to be investigated both in yeast and human cells.

In conclusion, both As(III) and Sb(III) appear to exhibit similar complex and indirect genotoxicity mechanisms, including replication-associated DNA damage mediated by oxidative stress and DNA–protein crosslinks as well as telomere dysfunction, which are exacerbated by metalloid-induced inhibition of key DNA repair pathways. Moreover, recent data obtained in a yeast model suggest that As(III) and Sb(III) may also have the ability to damage DNA independently of oxidative stress, replication or DNA–protein adducts. Although genotoxicity of As(III) at cytotoxic concentrations is evident in in vitro studies and in exposed humans, less is certain with regards to how As(III) contributes to carcinogenesis during long-term exposure to environmental relevant concentrations. As(III)-induced aneuploidy, which is also observed at low concentrations of As(III), may be another driving force of As(III)-induced genomic instability and also proteome imbalance. Low doses of As(III) inhibit expression and activity of key proteins involved in genome maintenance and cell cycle regulation, such as PARP-1 [111, 114–116] and p53 [162, 163]. In addition, different cell types or ages may vary in their telomere length, as well as their antioxidant, DNA repair, arsenic methylation and detoxification capacities, which may greatly influence susceptibility to As(III)-induced malignant transformation. Given the strong co-carcinogenic properties of As(III) and environmental pollution, co-exposure to other toxic metalloids and heavy metals, such as antimony and cadmium, and other pollutants, is also expected to promote carcinogenesis. Finally, it has recently been revealed that As(III) exhibits immunotoxic and immunosuppressive effects [164]. Some of these mechanisms may also be relevant for Sb(III), but both the genotoxic and carcinogenic properties of this metalloid require further extensive research.

## How arsenic causes proteotoxicity

Proteotoxic stress has emerged as an important contributor to the toxicity of arsenic. Traditionally, As(III) toxicity has been attributed to its interactions with sulfhydryl groups in folded native proteins, altering their function, regulation, and interactions [33, 36]. Recent studies revealed an additional mode of toxic action in which As(III) targets non-native proteins, thereby impairing their proper folding. Specifically, As(III) was shown to interfere with the refolding of chemically denatured proteins in vitro, with protein folding in vivo, and to cause misfolding and aggregation of nascent proteins in living cells.

**Fig. 2:**
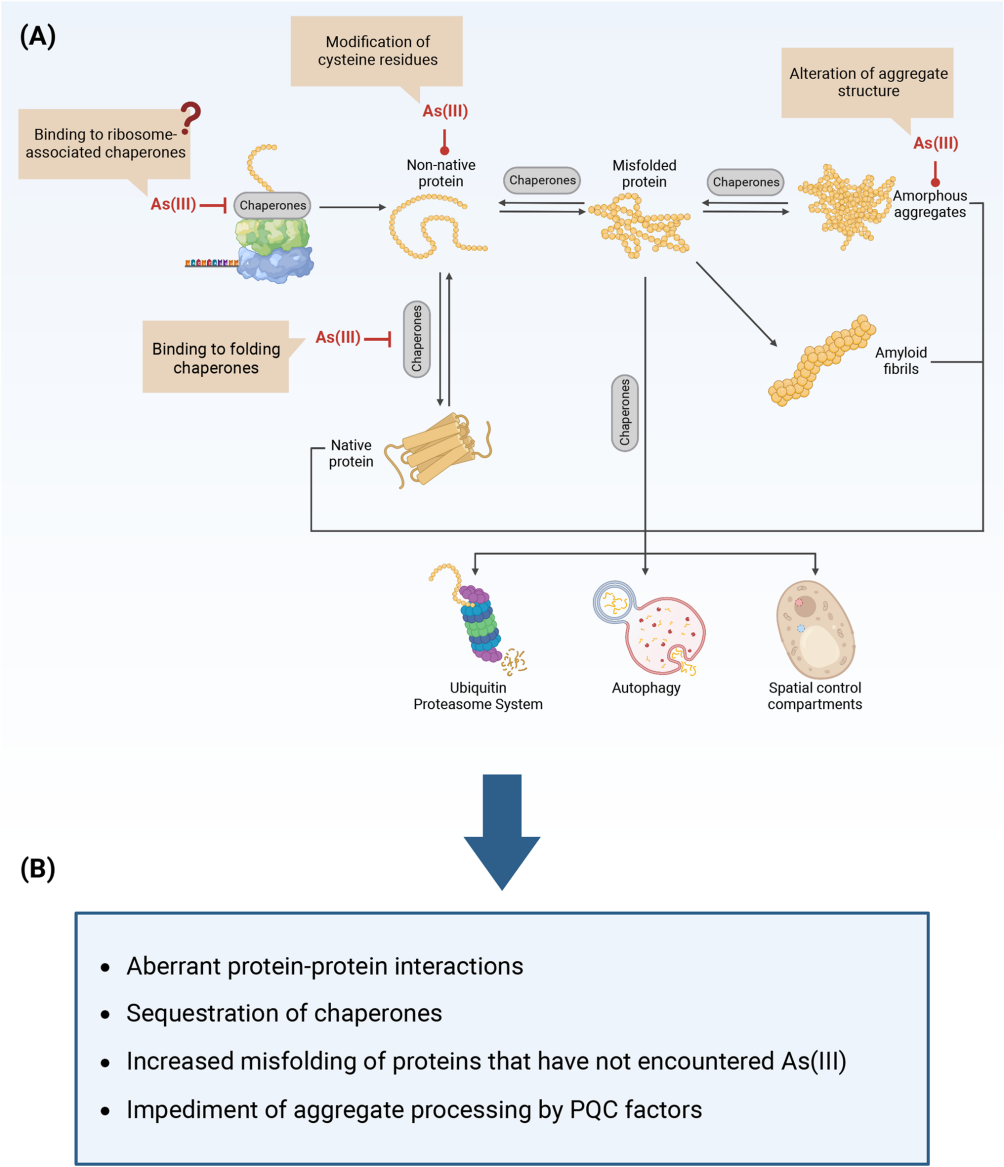
How arsenite causes proteotoxicity. **A** As(III) may bind to free thiols or other functional groups in nascent and non-native proteins, thereby preventing their folding into the native conformation, promoting protein misfolding and aggregation. As(III) may also impair chaperone-mediated folding and disaggregation by binding to the substrate protein, to molecular chaperones, and by modifying the structure of the aggregate. **B** The protein aggregates formed during As(III) exposure may contribute to toxicity by provoking aberrant protein–protein interactions, by sequestering chaperones, and by increasing the misfolding of other proteins that have not encountered the metalloid. Additionally, As(III) can affect aggregate structure such that processing by chaperones and possibly other PQC factors is impaired. The figure was created with BioRender.com

Protein folding and quality control is crucial for cell physiology and survival. To perform their biological function, most proteins must first adopt their native conformation, i.e., their folded three-dimensional structure. Protein folding takes place on the ribosome during translation, in the cytoplasm after ribosomal release, or in organelles such as the endoplasmic reticulum and mitochondria. If the correct native fold is not attained or lost, proteins can misfold and aggregate. Protein misfolding can occur due to mutations, external stress conditions, errors during transcription and translation, and during cellular aging [165, 166]. Protein misfolding and aggregation is detrimental for cells and organisms and is a feature of many human diseases including metabolic, oncological, and neurodegenerative disorders [167]. To ensure a functional proteome (protein homeostasis or proteostasis), cells use protein quality-control (PQC) systems (Fig. 2) that encompass (a) molecular chaperones that assist in the folding of proteins into their native conformation and in the disaggregation, refolding, sequestration, and degradation of non-native proteins; and (b) protein degradation systems such as the ubiquitin–proteasome system (UPS) and autophagy-lysosome pathway that eliminate misfolded and aggregated proteins [166, 168–170].

This section will highlight how protein aggregates are formed during As(III) stress, how these aggregates cause toxicity, and how cells maintain a healthy proteome during As(III) exposure. Most of the studies on metalloid-induced proteotoxicity described below have been performed in vitro and in model systems, primarily budding yeast and various human cell lines, whilst mechanistic studies in native cells and tissues are scarce.

### Misfolding and aggregation of nascent and non-native proteins is the prime mechanism of arsenite-induced proteotoxicity

A number of in vitro studies demonstrated that As(III) disrupts protein folding at µM concentrations. Initial studies indicated that the binding of As(III) to cysteine-containing model peptides perturbed their structures [171, 172]. Follow-up studies showed that As(III) inhibits oxidative refolding of certain disulfide-containing proteins (lysozyme, ribonuclease, riboflavin-binding protein) by binding to cysteine residues within the reduced and unfolded proteins [173, 174]. As(III) also interfered with spontaneous refolding of chemically denatured luciferase that possesses four cysteine residues but no disulfide bridge. Chaperone-mediated refolding of luciferase was also inhibited by As(III), but its impact was greater on the substrate than on the chaperone system [175]. Importantly, the defect in chaperone-mediated refolding of green fluorescent protein was abrogated in a cysteine-free version of the substrate protein [176], providing strong evidence that As(III) directly modifies cysteine residues in non-native target proteins. Together, these in vitro studies established that As(III) can inhibit the formation of the native protein fold by forming complexes with cysteine side chains (Fig. 2A).

As(III) has a strong impact on protein folding also in vivo. Studies in *S. cerevisiae* demonstrated that As(III) at concentrations ranging from 100 µM (subcytotoxic) to 1.5 mM (cytotoxic) provokes misfolding and aggregation of hundreds of cytosolic proteins and that aggregation is concentration-dependent [175–178]. Intracellular As(III) appears to be the culprit since overexpression of an As(III) export protein not only decreased intracellular arsenic concentrations but also protein aggregation levels [175]. Similarly, loss of yeast proteins that either restrict or enhance cytosolic arsenic concentrations is accompanied by reduced or increased protein aggregation levels, respectively, during As(III) stress [178].

What proteins are targeted by As(III) for misfolding and aggregation? Several observations indicate that As(III) primarily targets nascent or non-native proteins for misfolding and aggregation (Fig. 2A). In vitro experiments showed that As(III) has a modest impact on the activity of native luciferase whereas spontaneous and chaperone-mediated luciferase refolding were strongly inhibited [175]. In vivo experiments in yeast cells revealed that newly synthesized proteins aggregated during As(III) stress, and that As(III)-induced protein aggregation at concentrations of 100–500 µM was supressed when protein biosynthesis was inhibited, either chemically using a translation inhibitor [175] or genetically by deleting genes encoding proteins with functions in cytosolic translation [178, 179]. Proteomic studies further indicated that yeast proteins that are susceptible for aggregation during exposure to 1.5 mM As(III) have high translation rates and are substrates of ribosome-associated Hsp70 chaperones [175, 177, 180] while they appear relatively stable in their native (folded) state [177]. Collectively, these findings indicate that As(III) primarily impairs folding of nascent proteins rather than inducing large-scale unfolding of native proteins.

In addition to acting on non-native proteins, As(III) may also impair chaperone-assisted protein folding and disaggregation (Fig. 2A). In vitro experiments with the Hsp70 chaperone system of *Escherichia coli* (DnaK, DnaJ, GrpE) showed that As(III) interferes with chaperone-assisted refolding of denatured and heat-aggregated luciferase, even though the main impact of As(III) was on the substrate rather than on the chaperone system [175]. As(III) also inhibits substrate folding by the bovine and archaeal chaperonin TRiC/CCT in vitro [151]. As(III) was shown to bind to individual members of the human TRiC/CCT complex using a human proteome microarray [181] as well as to actin and tubulin in human lymphoblastoid cells and human breast cancer cells [145, 154] that are major TRiC/CCT substrates. Thus, it is not clear whether As(III) primarily affects TRiC/CCT activity or the folding substrate. Interestingly, the purified yeast Hsp70 and bichaperone Hsp70–Hsp104 systems were inhibited by As(III) to similar degrees as the bacterial Hsp70 system [175, 176]. However, instead of inhibiting the yeast chaperones, As(III) impaired protein disaggregation by modifying the structure of the aggregates, thereby preventing efficient binding of the chaperones to the aggregate [176]. One way As(III) could affect aggregate structure is by acting as an intermolecular crosslinker between two or three polypeptide chains leading to the formation of heterodimers and trimers [32, 36, 176]. Possibly, the observed changes of aggregate properties in the presence of As(III) [176] may not only reduce chaperone binding but might also affect aggregate processing by other PQC systems.

Experimental data support the notion that folding inhibition is an important contributor to the toxicity of As(III) that acts in parallel with other toxicity mechanisms. A genome-wide screen found a strong correlation between yeast mutants with enhanced protein aggregation levels and As(III) sensitivity, and between mutants with reduced aggregation levels and As(III) resistance [178]. Likewise, several studies in different model systems, including yeast (500 µM–1.5 mM), worm (*Caenorhabditis elegans*) (1.5–4.5 mM) and various mammalian cell lines (10–100 µM), indicated that maintaining proteostasis is crucial for cell survival and proliferation during As(III) exposure [175, 176, 178, 179, 182–187]. How aggregates formed during As(III) stress affect cell viability is not well understood. Protein aggregates can cause toxicity through multiple mechanisms. For example, aggregation could result in loss-of-function of individual proteins with protective or detoxification functions. Aggregation could also create a toxic gain-of-function where aggregated proteins damage membrane lipids, interact inappropriately with proteins and RNA, and sequester molecular chaperones and PQC factors [166]. Most likely, several mechanisms contribute to the toxicity caused by the aggregates formed during As(III) stress (Fig. 2B): (a) proteins that aggregate during As(III) stress are enriched for proteins with many interaction partners, suggesting that misfolded forms of these proteins could be involved in aberrant interactions [177]; (b) multiple chaperones associate with the aggregated proteins [175, 177], suggesting that chaperones may be sequestered away from the active pool of cytosolic PQC factors during As(III) stress; (c) in vitro experiments suggest that As(III)-aggregated protein seeds can drive misfolding and aggregation of other proteins that have not encountered the metalloid [175]; (d) aggregate size and structure may influence their seeding capacity and toxicity [166], and As(III) has been shown to modify the structure of aggregated model proteins [176, 188]. It will be important to elucidate the structure and properties of aggregates formed in the presence of As(III) to better understand their in vivo toxicity.

How does As(III) inhibit the folding of non-native proteins in vivo? On the molecular level, As(III) probably acts on not-yet folded segments of the polypeptide chain emerging from the ribosome during protein synthesis, preventing the formation of the native protein conformation. As(III) can bind to one, two or three thiol groups of cysteine residues with unidentate binding being weak whereas tridentate binding is highly stable [36]. Formation of stable pluridentate complexes between As(III) and exposed side chains is more likely to occur during the folding process when the protein backbone is flexible, and their formation prevents the protein from adopting its native conformation, promoting its misfolding and aggregation. In contrast, side chains are more likely to be fixed or buried inside the folded native protein, implying that formation of pluridentate complexes would require partial protein unfolding. We anticipate that As(III) affects folding of most nascent and non-native cellular proteins, and its impact on folding is likely influenced by the concentrations of both As(III) and the target proteins, the amino acid sequence of the target protein, the content and arrangement of thiol groups in side chains, the folding pathway, the rate of synthesis and folding, the structure of folding intermediates, and the efficacy of As(III) detoxification and PQC systems. While As(III) primarily targets cysteine-containing non-native proteins for aggregation, aggregation might also involve intermolecular hydrophobic interactions between As(III)-misfolded proteins and proteins that have not encountered the metalloid [32]. The observations that As(III)-aggregated proteins are enriched in multiple protein–protein interactions [177] and have seeding capacity [175], support this notion.

Additional ways arsenic could affect proteostasis and induce proteotoxicity include depletion of cellular ATP. In yeast, this mechanism appears unlikely for As(III), since As(III) concentrations that results in widespread protein aggregation in vivo (500 µM) had a negligible effect on intracellular ATP concentrations [176]. However, since ATP depletion can affect proteostasis [189], it is possible that As(V) could induce protein aggregation through disruption of ATP generation, although this remains to be demonstrated. Errors during transcription and translation may also lead to protein misfolding and aggregation [190, 191]. However, in budding yeast, 500 µM As(III) did neither induce errors during transcription [178], nor did it enhance mRNA mistranslation [175]. Finally, As(III) could affect proteostasis not only by stimulating the formation of protein aggregates, but also by interfering with their clearance through degradation. Studies in various mammalian cell lines showed that low As(III) concentrations (0.25–2 µM) can inhibit autophagic flux by blocking the autophagosome-lysosome fusion step through inhibition of SNARE complex formation [192, 193]. Whether this inhibition leads to the build-up of aggregates remains to be demonstrated. As(III) can bind to E3 ubiquitin ligases with RING finger domains [33, 194] and some studies using NIH3T3 and HEK293 cells suggested that arsenic (50–250 µM As(V), 1–10 µM As(III)) may inhibit the UPS [195, 196]. However, direct measurements of proteasomal activity revealed that, in fact, it increases in As(III)-exposed yeast (500 µM) and HeLa (25 µM) cells [175, 185, 197]. Nevertheless, it cannot be excluded that changes of aggregate structure by As(III) [176, 188] could affect aggregate processing and degradation.

Taken together, the impact of As(III) on non-native proteins, on chaperone machineries, and on aggregate structure and processing is expected to result in substantial protein misfolding and aggregation in cells, placing a heavy burden on the PQC systems.

### Do other metals and metalloids including antimonite disrupt protein folding in cells?

We predict that many more metals and metalloids have the potential to disrupt protein folding processes in living organisms [32]. Indeed, like As(III), cadmium [Cd(II)] induces misfolding and aggregation of nascent and non-native proteins in vivo. Interestingly, Zn(II) mitigated Cd(II)-induced protein aggregation in yeast cells [198], suggesting that Zn(II) may be substituted by Cd(II) while the nascent polypeptide chain undergoes the folding process [198, 199]. In contrast, Zn(II) did not alleviate As(III)-induced protein aggregation in yeast cells [198] even though As(III) can bind to ZnF motifs [37, 111]. Copper [Cu(I)] was shown to cause widespread protein aggregation in *E. coli*, probably by targeting cysteine- and histidine-containing proteins [200]. Whether Cu(I) primarily targets native or non-native proteins, remains to be determined. In case of chromium [Cr(VI)], protein aggregation in budding yeast is caused by enhanced mRNA mistranslation [201], but the underlying mechanism remains unknown. In vitro studies showed that mercury [Hg(II)] and lead [Pb(II)] can interfere with the refolding of chemically denatured proteins [202], but their effects on protein folding in vivo is not known. As outlined above, Sb(III) has high affinity for thiols and its toxicity may, in part, be attributed to its capacity to bind to cysteine residues in proteins [15, 42, 43, 136, 137]. Thus, it is conceivable that Sb(III) could disrupt protein folding in cells. A few studies suggest that Sb(III) might induce proteotoxic stress in cells: the molecular chaperones HSP70 and HSC70 were shown to confer Sb(III) tolerance to the protozoan parasite *Leishmania* [203] and work in yeast implicated the UPS in the protection from Sb(III) toxicity [182]. An in vitro study showed that Sb(III) can interact with and induce conformational changes in bovine serum albumin and to promote its partial aggregation [204]. Finally, antimony trioxide at high concentrations (400 µM–2.8 mM) induced transcription of genes encoding molecular chaperones as well as UPS- and autophagy-related genes in *Daphnia magna* [205]. Nevertheless, direct evidence that Sb(III) affects protein folding is currently lacking, which calls for more research in this area.

### Arsenite induces the formation of biomolecular condensates

As described above, As(III) can induce the formation of protein aggregates that are largely insoluble assemblies of misfolded proteins with aberrant and non-native conformations. Additionally, As(III) has been implicated in the formation of certain biomolecular condensates that are membraneless assemblies of proteins and nucleic acids. In contrast to aggregates, biomolecular condensates are characterized by weak, dynamic and multivalent interactions, are often formed via liquid–liquid phase separation, and carry out specialized functions in eukaryotic cells during physiological and stress conditions [206, 207]. Examples of condensates and their functions include stress granules (mRNA storage transcriptional regulation), processing bodies (mRNA decay and silencing), promyelocytic leukemia nuclear bodies (apoptotic signaling, anti-viral defence, DNA repair and transcription regulation), actin patches (endocytosis), heterochromatin (gene regulation), and nucleolus (ribosomal synthesis). Protein condensation and aggregation appear to be interlinked: (a) some condensates can transition from liquid-like states into more solid-like states when misfolded proteins associate with the condensates, (b) the cellular PQC machinery is implicated in the regulation of condensate formation, dissolution and dynamics, (c) a decline in PQC may contribute to the formation of aberrant, disease-causing condensates, and (d) there is evidence that certain condensates can serve as PQC compartments themselves [206, 208].

High concentrations of As(III) stimulates the formation of stress granules (SGs) and processing bodies (PBs) [209, 210] that are dynamic and reversible biomolecular condensates involved in mRNA storage and processing, and in transcriptional regulation [211]. Formation of SGs correlate with As(III)-induced translation repression through phosphorylation of the key translation initiation factor eIF2α [209, 212] and SG formation is important for stress adaptation [206, 211]. In contrast, PB formation does not involve eIF2α phosphorylation [209]. Work in *S. cerevisiae* suggests that the protein aggregates formed during As(III) stress are largely distinct from PBs and SGs as their localization and protein composition appear to differ to a large extent [175, 178]. Nevertheless, the PQC machinery is not only implicated in the processing of misfolded proteins but also in the regulation of SG and PB formation and dissolution, indicating cross-talk between these structures [206, 213].

Promyelocytic leukemia nuclear bodies (PML-NBs) are membraneless structures inside the nucleus involved in multiple genome maintenance pathways including the DNA damage response, DNA repair, telomere homeostasis, and p53-associated apoptosis [214]. The PML protein is the key driver of the formation of PML-NBs. The PML protein is also involved in the pathogenesis of APL and, as outlined above, arsenic trioxide can cure APL by binding to cysteine residues in the PML-RARα fusion protein inducing its oligomerization and subsequent degradation [37, 38]. Interestingly, As(III) stimulates PML-NB formation followed by PML degradation upon extended exposure in non-APL (HeLa) cells [215]. Additionally, PML-NBs have been proposed to have a function in nuclear protein homeostasis: upon proteotoxic stress, PML-NBs compartmentalize defective proteins and recruit chaperones and proteasomes to promote efficient degradation [216]. It is tempting to speculate that PML-NBs constitute a nuclear PQC compartment also during As(III) stress. In such a scenario, PML might act as an As(III) sensor that stimulates NB formation followed by recruitment of misfolded proteins and PQC components to restore proteostasis.

Other examples of condensates where misfolded proteins are stored, refolded or degraded during cellular stress conditions include the insoluble protein deposit (IPOD) and the juxtanuclear protein quality control (JUNQ) compartment [169, 206]. If and how As(III)-misfolded are partitioned into specific subcellular sites remains to be explored.

Recent studies indicate that DSB repair involves condensate formation [217]. Induction of DSBs triggers a local accumulation of DNA damage signaling and DNA repair proteins, called DDR foci, at sites of DNA breaks. Proper assembly and disassembly of DDR foci is crucial for rapid and efficient DNA repair coordinated with cell cycle arrest followed by recovery. Several DDR proteins have been shown to exhibit features of phase separation and to form condensates, such as the HR factor Rad52 in *S. cerevisiae* [218, 219] and the DNA damage signaling protein and DNA repair pathway choice regulator 53BP1 [220, 221] in human cells. It is believed that condensate formation may facilitate tethering of DNA molecules and control the recruitment and accumulation of DNA repair proteins at sites of DSBs or exclusion of other proteins [222]. For example, Rad52 condensates mediate nucleation of DNA damage-inducible intranuclear microtubule filaments, which facilitate Rad52 condensate fusion resulting in clustering of DSBs at the nuclear periphery for repair [218]. Interestingly, proteins condensates have also been implicated in polymerization, organization and dynamics of both tubulin and actin cytoskeleton [223–225]. 53BP1 condensates act as a scaffold for the tumor suppressor protein p53, promoting its activation, thereby inducing changes in transcription of p53-targeted genes and checkpoint activation in response to DNA damage [220]. On the other hand, 53BP1 is excluded from the PAR-seeded condensates of the low complexity domain-containing FET proteins (FUS, EWS, TAF15) [226]. FET condensates are an early but transient event at DNA damage sites and can therefore control the spatiotemporal assembly and disassembly of DDR proteins. A recent in vitro study demonstrated that telomeres also undergo a phase separation-driven compartmentalization that regulates the access of telomere-associated and DNA repair proteins to chromosome ends [227]. Considering the observed negative effects of As(III) and Sb(III) on DSB repair, PARP-1-mediated PAR synthesis, cytoskeleton morphology and telomere stability, it is tempting to speculate that the mechanisms of metalloid toxicity may also involve disruption of biomolecular condensate-driven cellular processes by interfering with post-translation modifications and/or folding of proteins involved in condensate formation.

Regulation of eukaryotic gene expression has been shown to involve condensate formation of gene-specific transcription factors with coactivator and RNA polymerase II complexes [228–230]. Whether metalloids promote condensate formation of specific transcription factors involved in detoxification and defence remains unknown.

To conclude, recent studies established that the prime mechanism of As(III)-induced proteotoxicity is governed by interactions between As(III) and exposed cysteine residues in nascent or non-native proteins. This interaction obstructs the formation of the native protein conformation and promotes protein misfolding and aggregation. Additionally, As(III) may potentially regulate or disrupt formation of biomolecular condensates as a toxicity or defence mechanism. Due to the importance of biomolecular condensates in health and disease, future research efforts should be directed toward the elucidation of how arsenic, antimony and other metals affect condensate biogenesis.

### Mechanisms that protect cells from metalloid-induced protein misfolding and aggregation

Cells rely on two main strategies to maintain a functional proteome during As(III) exposure. The first strategy relies on damage prevention. Cells can protect nascent proteins from harmful arsenic interactions by limiting intracellular arsenic concentrations. Yeast cells respond to As(III) by regulating influx, efflux, and sequestration systems [46, 51, 231–234], and this response is important to safeguard proteostasis: yeast cells capable of restricting cytosolic arsenic concentrations show reduced protein aggregation levels whilst a failure in limiting cytosolic arsenic is accompanied by enhanced protein aggregation [178]. Regulation of certain arsenic influx and efflux systems involves the transcription factor Yap8 and the MAP kinase Hog1 that act as direct sensors of intracellular As(III) [39, 41, 51]. Thus, these proteins couple arsenic-binding to improved proteostasis and cell survival. Intracellular As(III) chelation by GSH also protects cells from extensive protein aggregation, probably by lowering the cytosolic concentration of ‘free’ As(III) that can interfere with protein folding processes [233].

Another way of preventing or reducing aggregate formation is to repress translation. A number of studies in yeast (500 µM–1.5 mM) and human cell lines (10–150 µM) demonstrated that As(III)-exposed cells repress global protein biosynthesis, probably as a result of increased phosphorylation of the translation initiation factor eIF2α and subsequent inhibition of translation initiation [178, 179, 183, 187, 235, 236]. Additionally, studies in yeast cells showed that expression of genes encoding aggregation-prone proteins [177] and of protein biosynthesis–related genes [183, 234, 237] were repressed in response to As(III) exposure. Translation repression is crucial for maintaining proteostasis during As(III) stress, since yeast mutants with low global translation activity as well as mutants with improved translation repression efficiency are less susceptible for As(III)-induced protein aggregation and toxicity [178, 179]. Conversely, yeast mutants that are defective in translation repression show enhanced protein aggregation levels and As(III) sensitivity [178]. The importance of translation control during As(III) stress is further emphasized by the observation that deletion of yeast genes encoding functions in protein biosynthesis leads to reduced protein aggregation and As(III) resistance [151, 178, 179, 183]. Together, these studies establish that an accurate control of protein synthesis is of central importance to ensure proteostasis and cell viability during As(III) stress, probably by lowering the influx of misfolded proteins into the proteome. Little is known about the sensing and signaling mechanisms that regulate translation during As(III) stress, although the heme-regulated inhibitor kinase (HRI) in mouse cells [187] and the TORC1 (TOR complex 1) protein kinase in yeast [238] have been implicated.

The second strategy relies on damage elimination. Studies in various cell types and model organisms have shown that expression of genes encoding functions in protein folding and degradation is induced in response to As(III) (concentrations ranging from 0.5 to 50 µM for mammalian cells; 0.5–1.5 mM in yeast) [46, 175, 185, 196, 234, 237, 239–242]. Expression of genes related to ATP production is also induced by As(III) [234, 237], and yeast cells ensure that sufficient ATP is available for their protein folding and degradation needs [176]. Protein aggregates formed during As(III) stress are preferentially degraded through the UPS (Fig. 2A). Studies in budding yeast showed that the aggregates are tagged with K48-linked ubiquitin chains [176, 179] and that cells increase the amount of proteasomal components as well as proteasomal activity during As(III) stress [175, 183, 234, 237]. Inhibition of proteasomal activity by genetic or chemical means impairs the clearance of aggregates [175, 176, 178] and sensitizes yeast cells to As(III) [175, 176, 182, 183]. Similarly, mouse fibroblasts and HeLa cells exposed to 25 µM As(III) accumulate K48-linked ubiquitin conjugates and increase proteasomal activity [185, 197]. Interestingly, inhibition of protein ubiquitination abrogates As(III)- and heat-induced stimulation of proteasomal activity in HeLa cells, suggesting that proteasomal activation may be signaled by the build-up of ubiquitinated proteins in the cells [197]. Studies in mouse cells and *C. elegans* indicated that proteasomes may adapt to As(III) stress via the arsenite-inducible RNA-associated protein (AIRAP), that is induced by As(III) and associates with proteasomes to regulate their degradative capacity [185]. Yeast cells also possess As(III)-inducible components of the proteasome including Cuz1 (ZFAND1 in mammalian cells) and Tmc1 (an AIRAP orthologue). Yeast Cuz1 and mammalian ZFAND1 interact with the proteasome and with Cdc48 (p97 in mammalian cells), an ATPase that delivers proteins to the proteasome for degradation, while Tmc1 and AIRAP associate with the proteasome’s 19S cap [182, 243, 244]. Loss of either Cuz1 or Tmc1 sensitizes yeast cells to As(III) and Sb(III) [182]. Similarly, loss of AIRAP sensitizes worms to As(III) [184]. Whether cells lacking these regulators accumulate protein aggregates during As(III) stress remains to be determined. Moreover, it is currently not known how these proteins regulate proteasomal activity during As(III) stress or if and why they are specifically required during proteotoxic stress caused by As(III). One possibility is that changes in aggregate structure inflicted by As(III) [176, 188] necessitates an adaptation of the proteasome for efficient substrate degradation. In addition to K48-linked chains, the aggregates formed during As(III) stress are also tagged with K63-linked chains [179]. K63-linked chains have primarily been associated with proteasome-independent processes such as DNA repair, endocytosis and selective autophagy [245, 246] but have recently been shown to play a role in proteasome-dependent substrate degradation when forming branched K48/K63 chains [247, 248]. Since branched ubiquitin chains are stronger degradation signals than unbranched chains [247, 248], the presence of both K48- and K63-linked chains on As(III) aggregated proteins raises the possibility that these aggregates may be difficult substrates for the proteasomes. Nevertheless, whether the K48 and K63 chains assembled on As(III)-aggregated proteins are homotypic or heterotypic branched K48/K63 chains remains to be demonstrated. Similarly, the ubiquitin ligases that mark the proteins that misfold during As(III) stress with K48 and K63-linked chains, remain to identified.

In addition to the UPS, the autophagy–lysosome pathway also contributes to aggregate clearance and As(III) resistance (Fig. 2A). Autophagy is activated in yeast (0.5–1.5 mM) and human bronchial epithelial cells (0.25 µM) exposed to As(III) [183, 249] and several yeast mutants lacking autophagy–lysosome pathway components have increased levels of protein aggregates during As(III) stress [176, 178]. While these findings implicate autophagy in the clearance of As(III)-induced aggregates, autophagy appears less prominent than the UPS [176, 183]. The weaker contribution of autophagy to aggregate clearance might be due that fact that As(III) can inhibit autophagic flux [192, 193] or that the two pathways recognize distinct subsets of misfolded and aggregated substrates.

The cellular concentration of molecular chaperones increases in response to As(III). Molecular chaperones are implicated in the folding, sorting, disaggregation, and degradation of proteins [166, 168, 169] implying that their upregulation is important to meet an increased protein folding, sorting, and/or degradation demand. Several chaperones co-sediment with aggregated proteins during As(III) stress in vivo [175], suggesting that they are engaged in the disaggregation, refolding or degradation of substrate proteins. Alternatively, these chaperone interactions might be important to prevent As(III)-misfolded proteins from erroneous interactions with other proteins. Chaperone-mediated disaggregation has been implicated in aggregate clearance: deletion or chemical inhibition of the yeast disaggregase Hsp104, its cytosolic co-chaperones Ydj1 (Hsp40) or Ssa1 and Ssa2 (Hsp70) delayed the clearance of protein aggregates formed during As(III) stress [176]. The fate of the proteins recovered from the aggregates is, however, not entirely clear. Heat-aggregated proteins are preferentially refolded rather than degraded in yeast [250] and thermotolerance is strongly dependent on Hsp104 [251]. In contrast, yeast cells lacking Hsp104 are not sensitive to As(III) at concentrations that provoke protein misfolding and aggregation [176, 252]. The observation that aggregates formed in the presence of As(III) are poor substrates for chaperone binding [176] might explain why Hsp104 is largely dispensable for growth and survival during As(III) stress. Moreover, overexpression of Hsp104 sensitizes yeast cells to As(III) [176], possibly due to high unfolding activity [253] and subsequent build-up of misfolded proteins [176]. These observations suggest that the prime fate of As(III)-misfolded proteins might be their degradation rather than refolding. Indeed, yeast Ydj1 and Ssa1/Ssa2 are not only involved in protein folding but also in protein turnover, keeping misfolded substrates soluble until they are ubiquitinated by ubiquitin ligases for subsequent degradation [254–257]. The GimC/prefoldin chaperone complex is also implicated in aggregate clearance during As(III) stress in yeast [179]. Similar to Hsp40 and Hsp70, GimC/prefoldin is primarily involved in nascent protein folding [258] but has also been implicated in protein turnover, facilitating degradation by maintaining substrate solubility [259]. Thus, molecular chaperones may be important to keep As(III)-misfolded proteins soluble, promoting their ubiquitination and degradation. Molecular chaperones may also direct misfolded proteins to specific subcellular sites, such as the IPOD and JUNQ, as a means to protect the intracellular environment [166, 169, 260–263]. Whether the aggregates formed during As(III) stress are sequestered to specific sites within the cell remains unknown.

## Metalloid-induced genotoxicity and proteotoxicity: a double-edged sword targeting neurodegeneration

A common denominator of many age-related and neurodegenerative diseases is the dysfunction of proteostasis and the pathological accumulation of protein aggregates. Proteins that adopt non-native conformations in neurodegenerative diseases include amyloid β (Aβ) and tau in Alzheimer’s disease (AD), α-synuclein (αSyn) in Parkinson’s disease (PD), TAR DNA binding protein 43 (TDP-43) and RNA/DNA-binding protein fused in sarcoma (FUS) in amyotrophic lateral sclerosis (ALS) and frontotemporal lobar degeneration (FTLD), and huntingtin (HTT) in Huntington disease (HD) [167, 264, 265]. Epidemiological studies have indicated an association between arsenic exposure and the prevalence of several neurodegenerative disorders, including AD and PD [13, 266–270]. However, the mechanisms by which arsenic contribute to these proteinopathies are poorly understood. Arsenic is a well-established neurotoxin that impairs cognitive functions and memory, especially in children, deteriorates mental health, and causes peripheral neuropathy. Several of these effects have been replicated in rodent studies [271, 272]. At the molecular level, it is believed that the neurotoxic properties of As(III) mainly stem from mitochondrial dysfunction and oxidative stress-derived cellular damage, disruption of proteostasis, neuroinflammation and neuronal apoptosis [13, 268]. Arsenic (and perhaps also antimony) can induce proteotoxicity and several yeast proteins that aggregate in As(III)-exposed cells have human or mouse orthologues that are implicated in proteinopathies and/or co-aggregate with disease-associated proteins in AD, familial ALS or PD [177]. Thus, metalloid-induced and disease-associated protein aggregation may have some shared features.

PD is characterized by the accumulation of αSyn aggregates in the neurons of the *substantia nigra pars compacta*, leading to neuronal cell death and, consequently, neurodegeneration [273]. Studies in neuroblastoma cell lines, rats, and mice revealed a time-dependent accumulation and oligomerization/aggregation of αSyn during exposure to low As(III) concentrations (0.03–0.3 µM) [274, 275]. Chronic exposure of mice to environmental relevant (0.65–6.5 μM) and high (65 μM) concentrations of As(III) in drinking water caused a dose-dependent increase in the phosphorylation of leucine-rich repeat kinase 2 (LRRK2) and αSyn in different brain regions [276]. Both LRRK2 and αSyn phosphorylation are implicated in PD, and phosphorylation is known to modulate αSyn oligomerization and fibrilization, as well as the formation of Lewy bodies and neurotoxicity [277, 278]. Several metals can affect αSyn conformation upon binding [279–282] and in vitro data indicate that As(III) may be incorporated into and alter the structure of amyloid fibres formed by acetylated human αSyn [188]. The presence of As(III) also affects the intracellular distribution and clearance of αSyn aggregates and aggravates αSyn toxicity in yeast cells [188]. Together, these observations suggest that A(III) may influence αSyn conformation, phosphorylation, regulation, and toxicity.

AD is characterized by insoluble deposits of various Aβ peptides, generated by cleavage of the β-amyloid precursor protein (APP), and of phosphorylated tau protein [283, 284]. Exposure to 5 μM As(III) and 10 μM DMAs(III) results in elevated APP levels in cholinergic cells. While DMAs(III) also increased Aβ formation, Aβ levels were unchanged or even reduced during As(III) stress [285]. However, when combined with Cd(II) and Pd(II), 5–50 μM As(III) greatly increased Aβ formation in rats, which was accompanied by cognitive impairments [286]. Additionally, formation of Aβ aggregates was stimulated by chronic exposure to As(III) (40 μM) in a mouse model of AD [287]. Together, these studies indicate that As(III) has the capacity to induce Aβ aggregation. As(III) has been shown to activate the tau kinases, such as GSK3, ERK1/2, JNK, and CDK5 in human neuroblastoma SH-SY5Y cells (5–10 μM) [287, 288] and in the rat brain (intraperitoneal injections of 2.5 mg/kg body weight for 28 days) [287–290] and consequently leading to hyperphosphorylation of several tau residues, which are also hyperphosphorylated under pathological conditions. As(III) (1–20 µM) also increased the formation of tau aggregates in human neuroblastoma SH-SY5Y cells at concentrations of 5–10 μM [288], consistent with the view that tau hyperphosphorylation leads to the formation of oligomers and neurofibrils [291, 292]. Thus, As(III) may induce tau aggregation by affecting its phosphorylation state.

Not much is known about the neurotoxic potential of antimony. Recent studies in mice revealed that Sb(III) exposure (10–40 mg/kg body weight) resulted in impaired learning and spatial memory [293] and neurotoxicity via ROS-mediated autophagic death of neurons [294]. Interestingly, long-term exposure of mice to Sb(III) (10–20 mg/kg body weight) triggered tau hyperphosphorylation and accumulation of tau and Aβ deposits [295]. Hence, Sb(III) exposure might be a risk factor for AD and other neurodegenerative diseases.

**Fig. 3:**
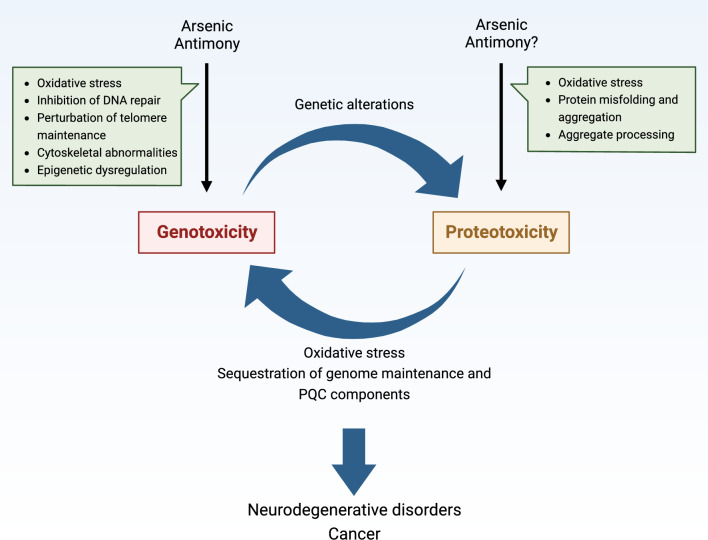
How metalloid-induced genotoxicity and proteotoxicity may be interrelated. Arsenic and antimony may induce genotoxicity through oxidative stress, inhibition of DNA repair, perturbation of telomere maintenance, cytoskeletal abnormalities, and epigenetic dysregulation. Arsenite (and perhaps also antimonite) may cause proteotoxicity through oxidative stress, protein misfolding and aggregation and defective aggregate processing. Metalloid-induced genetic alterations may generate proteotoxic stress whereas protein aggregation may lead to enhanced oxidative stress and sequestration of genome maintenance and protein quality-control (PQC) components. Thus, metalloid-induced genotoxicity and proteotoxicity may be interrelated, where loss of genome integrity amplifies the impact of metalloids on proteome integrity and vice versa. In this way, genome instability and proteome instability are interrelated and jointly contribute to neurodegeneration and carcinogenesis. The figure was created with BioRender.com

The above studies suggest that metalloids like As(III) and Sb(III) may contribute to proteinopathies by inducing misfolding and aggregation of specific disease-associated proteins such as αSyn, Aβ, and tau, by interacting with the protein, by inducing post-translational modifications such as phosphorylation, and/or via other mechanisms such as induction of oxidative stress (Fig. 3). Moreover, the strong impact of As(III), and perhaps also Sb(III), on global protein misfolding that propels extensive protein aggregation in cells, may also play a part in the initiation or progression of degenerative diseases. These effects are likely more severe with increasing age when the efficacy of the PQC systems decline.

It is important to note that beside pathological protein aggregation and aberrant proteostasis, neurodegenerative disease can arise due to several other mechanisms including DNA and RNA defects, neuronal cell death, synaptic and neuronal network defects, cytoskeletal abnormalities, and altered energy homeostasis [265]. In recent years, it has become increasingly clear that genome instability is another important contributor to neurodegenerative disorders [296] as well as cancer [297] and aging [298]. For example, AD, PD, ALS, HD, and FTLD are not only characterized by the accumulation of pathological protein aggregates, but also by increased levels of DNA damage [299–302]. A growing body of evidence suggests that age-related mitochondrial dysfunction and increased oxidative stress, accompanied by compromised DNA repair, result in genome instability that may precede and/or exacerbate proteome instability in neurons [303–307]. Several genetic disorders caused by mutations in genes encoding DNA damage sensors and DNA repair proteins also lead to neurodegenerative pathologies [308, 309]. Furthermore, loss or inhibition of human DNA damage sensor proteins, such as ATM, ATR, and MRE11, as well as induction of genotoxic stress in form of topoisomerase-DNA adducts, result in widespread protein aggregation [310–312]. Similarly, the budding yeast ATR orthologue Mec1 confers resistance to proteotoxic stress, suggesting an evolutionarily conserved role of the DNA damage response signaling pathway in proteostasis [313].

Notably, several proteins that form pathological aggregates have functions in DNA metabolism and genome maintenance. It is believed that cytosolic aggregation of these proteins may impair their nuclear functions in preserving genomic integrity of neurons, leading to build-up of DNA damage and induction of regulated cell death. For example, TDP-43, which exhibits nuclear exit and cytosolic aggregation in motor neurons in tau-negative FTLD patients and in 95% of ALS patients [314], is involved in DSB repair by NHEJ [305]. Loss of TDP-43 nuclear localization results in persistent DNA damage and prolonged activation of DNA damage response signaling, ultimately leading to neuronal death [305]. FUS, whose defects are associated with ~ 5% of familial ALS and ~ 1% of sporadic ALS cases due to loss of its nuclear localization and subsequent cytosolic aggregation, has been shown to facilitate DNA ligation during the final step of oxidative damage repair [315] and to be required for DSB repair by NHEJ and HR [316, 317]. HD-associated wild-type HTT is recruited to sites of DNA damage, colocalizes with BER proteins during oxidative stress [318], and serves as a scaffold protein for the transcription-coupled DNA repair (TCR) complex [319]. αSyn forms nuclear foci, which colocalize with γH2AX, and enhances ligation of DNA ends in vitro, and repair of bleomycin-induced DSBs is compromised in human HAP1 cells with αSyn knock-out [320]. Tau is involved in the organization of the microtubule cytoskeleton and the regulation of protein trafficking on microtubules [321], and may have a general role in microtubule-mediated transport of DNA repair proteins into the nucleus, as shown for the 53BP1 protein required for NHEJ [160, 322]. Tau is also implicated in the protection of neuronal DNA by an as yet poorly understood mechanism [323].

At the same time, protein aggregates may directly or indirectly cause DNA damage. Using in vitro models of PD, it was shown that αSyn overexpression induces DNA breaks in the presence of pro-oxidant Fe salts and that the DNA nicking property of αSyn is enhanced by its misfolding [324]. Pre-formed fibrils (PFF) of αSyn activates NOS, leading to oxidative DNA damage and PARP-1 activation. Excessive PAR levels, as well as PARylation of αSyn PFF, not only accelerates αSyn aggregation but also leads to neuronal cell death via parthanatos [325]. Interestingly, lack of ATM in concert with elevated ROS triggers the accumulation of transcription-dependent SSBs followed by increased protein PARylation at sites of DNA damage and subsequent protein aggregation [311]. Consequently, PARP-1 inhibition shows neuroprotective effects not only in PD [325] but also in ALS [326] and HD [327]. A recent study showed that αSyn aggregation probably induces DSBs, as indicated by increased formation of γH2AX foci, both in mixed glial cultures and in the αSyn PFF mouse model of PD. This is followed by activation of the cyclic GMP–AMP synthase (cGAS)/stimulator of interferon genes (STING), resulting in neuroinflammation that is known to contribute to neurodegeneration in PD [302]. Protein aggregation may also negatively affect DNA repair pathways indirectly by decreasing the levels of DDR proteins or by sequestering DDR proteins in the cytoplasm of neurons. For example, BRCA1, a key factor in DSB repair by HR, is sequestered into cytosolic inclusions of hyperphoshorylated tau in AD brain samples [328], while reduced BRCA1 levels are observed in neurons exposed to Aβ oligomers and in AD patients [303].

Another source of protein aggregate-derived DNA damage could be ROS. It is well established that mitochondrial dysfunction and increased oxidative stress contribute to neurodegenerative disorders and aging of the human brain [329–331]. Elevated oxidative damage to mitochondrial and nuclear DNA was observed in brain tissue from AD, PD, HD, and ALS patients and rodent models [309, 332, 333]. The origin of increased ROS production and the mechanisms underlying redox imbalances remain elusive, but most likely involve physical interactions of protein aggregates with mitochondria, leading to impaired mitochondrial metabolism [309, 330, 331, 333], and/or aggregation of mutant superoxide dismutase SOD1, as observed in some ALS cases, resulting in nuclear depletion of wild type SOD1 [334].

Taken together, accumulating lines of evidence suggest that genome instability and proteome instability are interrelated and jointly contribute to neurodegeneration and carcinogenesis (Fig. 3). Since cells exposed to As(III) and Sb(III) suffer from both genotoxic and proteotoxic damage, the possibility that both modes of toxic action are mechanistically related and together contribute to neurotoxicity and the development of neurodegenerative disorders should be considered. Importantly, the negative effects of metalloids on cells, including neurons, largely overlap with hallmarks of neurodegeneration. The genotoxic properties of metalloids, such as oxidative stress-derived DNA damage, inhibition of DNA repair mechanisms, induction of topoisomerase–DNA adducts, and perturbation of the cytoskeleton, may lead to genome instability and subsequent overload of PQC systems and increased protein aggregation, as observed under various genotoxic conditions [312]. At the same time, metalloids induce pathological protein aggregation as well as global protein misfolding and aggregation, which may also negatively impact the PQC systems and further promote genome and proteome instability. Thus, the genotoxic and proteotoxic effects of metalloids may act as a double-edged sword with significant potential to both initiate and accelerate neurodegeneration.

## Conclusions and future perspectives

In this review, we highlighted how the metalloids arsenic and antimony cause genotoxicity and proteotoxicity and presented evidence that they may contribute to disease in a concerted way. Our understanding of how metalloids cause genotoxic and proteotoxic stress and the ensuing human health impacts, has increased considerably in the past decades. However, several key aspects of metalloid toxicity and cellular defence remain incompletely understood. For example, an important area for future studies is to clarify aggregate toxicity mechanisms, specifically the structures and properties of the protein aggregates formed during metalloid exposure that contribute to their toxicity as well as the features of these aggregates that limit aggregate binding and processing by chaperones and perhaps other PQC factors. A second key question concerns sensing and signaling mechanisms, specifically how cells detect metalloids and/or metalloid-induced cellular damage and how they couple the sensing mechanisms to the regulation of protective systems. The third question concerns the generality of the observations that metals and metalloids can perturb protein folding in cells, specifically which metals affect proteostasis in vivo and the mechanisms whereby the cause proteotoxicity. Loss of proteome and genome integrity are common hallmarks of neurodegenerative diseases, aging, and cancer. Given that certain metals and metalloids can cause proteotoxic and genotoxic effects, suggest that metals may be major environmental factors driving these diseases. Thus, future efforts should be directed toward elucidating mechanisms by which metalloids initiate or accelerate neurodegeneration and other human diseases. Similarly, how metal/metalloid-induced genotoxic and proteotoxic stress are interrelated and jointly contribute to neurodegeneration and carcinogenesis remains to be clarified. Finally, epidemiological studies are needed to cement the importance of individual metals as risk factors for disease, and regulatory measures should be taken to reduce exposure levels. Molecular insights into cellular toxicity and response mechanisms and their links to pathogenesis especially at low environmentally-relevant concentrations, may pave the way for the development of strategies for both disease prevention and treatment.

## Data Availability

Not applicable.

## Abbreviations

8-OHdG: 8-Hydroxyl-2′-deoxyguanine
Aβ: Amyloid β
ABC: ATP-binding cassette
AD: Alzheimer’s disease
AIRAP: Arsenite-inducible RNA-associated protein
αSyn: α-Synuclein
ALS: Amyotrophic lateral sclerosis
APL: Acute promyelocytic leukemia
APP: Amyloid precursor protein
As(V): Arsenate
As(III): Arsenite
ATP: Adenosine triphosphate
BER: Base excision repair
cGAS: Cyclic GMP–AMP synthase
DDR: DNA damage response
DDT: DNA damage tolerance
DMAs(III): Dimethylarsonous acid
DSB: Double-strand break
FET: FUS, EWS, TAF15
FTLD: Frontotemporal lobar degeneration
FUS: Fused in sarcoma
GSH: Reduced glutathione
GSSG: Oxidized glutathione
GTP: Guanosine triphosphate
HD: Huntington disease
HR: Homologous recombination
HRI: Heme-regulated inhibitor kinase
hTERT: Human telomerase reverse transcriptase
Htt: Huntingtin
ICR: Interstrand crosslink repair
IPOD: Insoluble protein deposit
JUNQ: Juxtanuclear protein quality control
LRRK2: Leucine-rich repeat kinase 2
MMAs(III): Monomethylarsonous acid
MMR: Mismatch repair
NER: Nucleotide excision repair
NHEJ: Non-homologous end joining
NOS: Nitric oxide synthase
NOX: NADPH oxidase
PAR: Poly(ADP-ribose)
PARP-1: Poly(ADP-ribose) polymerase-1
PB: Processing body
PD: Parkinson’s disease
PFF: Pre-formed fibrils
PML-NBs: Promyelocytic leukemia nuclear bodies
PQC: Protein quality control
RING: Really interesting new gene
RNS: Reactive nitrogen species
ROS: Reactive oxygen species
SAC: Spindle assembly checkpoint
Sb(III): Antimonite
SG: Stress granule
SSB: Single-strand break
STING: Stimulator of interferon genes
TCR: Transcription-coupled DNA repair
TDP-43: TAR DNA binding protein 43
TLS: Translesion synthesis
Top1: DNA topoisomerase I
TOP1cc: DNA topoisomerase 1 cleavage complex
TORC1: TOR complex 1
UPS: Ubiquitin–proteasome system
UVC: Ultraviolet C radiation
WHO: World Health Organization
XPA: Xeroderma pigmentosum complementation group A protein
ZnF: Zinc finger
